# Bioinspired robots can foster nature conservation

**DOI:** 10.3389/frobt.2023.1145798

**Published:** 2023-10-18

**Authors:** Mrudul Chellapurath, Pranav C. Khandelwal, Andrew K. Schulz

**Affiliations:** ^1^ Max Planck Institute for Intelligent Systems, Stuttgart, Germany; ^2^ KTH Royal Institute of Technology, Stockholm, Sweden; ^3^ Institute of Flight Mechanics and Controls, University of Stuttgart, Stuttgart, Germany

**Keywords:** biomimetics, bioinspiration, collaboration, locomotion, exploration, monitoring, intervention, maintenance

## Abstract

We live in a time of unprecedented scientific and human progress while being increasingly aware of its negative impacts on our planet’s health. Aerial, terrestrial, and aquatic ecosystems have significantly declined putting us on course to a sixth mass extinction event. Nonetheless, the advances made in science, engineering, and technology have given us the opportunity to reverse some of our ecosystem damage and preserve them through conservation efforts around the world. However, current conservation efforts are primarily human led with assistance from conventional robotic systems which limit their scope and effectiveness, along with negatively impacting the surroundings. In this perspective, we present the field of bioinspired robotics to develop versatile agents for future conservation efforts that can operate in the natural environment while minimizing the disturbance/impact to its inhabitants and the environment’s natural state. We provide an operational and environmental framework that should be considered while developing bioinspired robots for conservation. These considerations go beyond addressing the challenges of human-led conservation efforts and leverage the advancements in the field of materials, intelligence, and energy harvesting, to make bioinspired robots move and sense like animals. In doing so, it makes bioinspired robots an attractive, non-invasive, sustainable, and effective conservation tool for exploration, data collection, intervention, and maintenance tasks. Finally, we discuss the development of bioinspired robots in the context of collaboration, practicality, and applicability that would ensure their further development and widespread use to protect and preserve our natural world.

## 1 Introduction

Humans have arrived at a critical juncture in their relationship with nature. Human activities such as unsustainable resource extraction, large-scale production/development, and air, water, and land pollution are degrading the planet’s health and threatening the existence of its inhabitants (Visbeck, 2018). The last 50 years have seen an exponential decline in ecosystem health and a loss of nearly 70% of our planet’s biodiversity (Ledger et al., 2023). Conservation efforts are critical to protecting and reviving ecosystems around the world and to prevent a sixth-mass extinction event (Hendriks et al., 2006; Ceballos et al., 2015).

Conservation efforts can be broadly divided into exploration, data collection, monitoring, intervention, and maintenance tasks. Each of these tasks is essential to address ecosystem knowledge gaps, promote ecosystem health, and work towards reversing the ecosystem damage (Table 1). For example, an estimated 17,000 out of 120,000 species monitored by the IUCN Red List of Threatened Species are listed as ‘Data deficient’, meaning there is not enough data to make a reliable population estimate (Tuia et al., 2022). Moreover, a vast majority of the oceans’ depths are still unexplored, and the human impact on them remains unknown (Roberto et al., 2020). These ecosystem knowledge gaps emphasize the need for innovative and efficient approaches for conducting exploration, data collection, and monitoring activities. Equally important in conservation efforts are intervention and maintenance tasks. Intervention tasks such as tackling the spread of invasive species are required to prevent biodiversity loss and maintain healthy functioning of the ecosystem (Linders et al., 2019). Additionally, the proper management of invasive species can lead to overall economic benefits for the local communities (Keller et al., 2008; Hanley and Roberts, 2019). Maintenance tasks including removal of trash generated by humans or managing insect outbreaks and forest fires are critical to ensure the continuity of ecosystem services (Daily, 1997; Amelia et al., 2021; Gross, 2021).

**TABLE 1:** Brief description of tasks involved during conservation.

Conservation task	Broad definition
Exploration	Accessing terrestrial, marine, arctic, or aerial environments to document known and unknown biodiversity, including previously unexplored environments
Data collection	Engaging in invasive or non-invasive sample collection and/or information gathering to address specific questions concerning the ecosystem
Monitoring	Regular surveillance to assess the climate and ecosystem state (e.g., population, invasive species, human-wildlife interactions, and overall ecosystem health)
Intervention	Undertaking activities that promote ecosystem recovery and improve it’s overall state (e.g., re-wilding, reintroduction of focal species, and removal of invasive species)
Maintenance	Undertaking activities that preserve the present natural state of the ecosystem, and ensure sustainability of ecosystem services (e.g., tackling disease outbreaks, natural disasters, and human trash)

**FIGURE 1:**
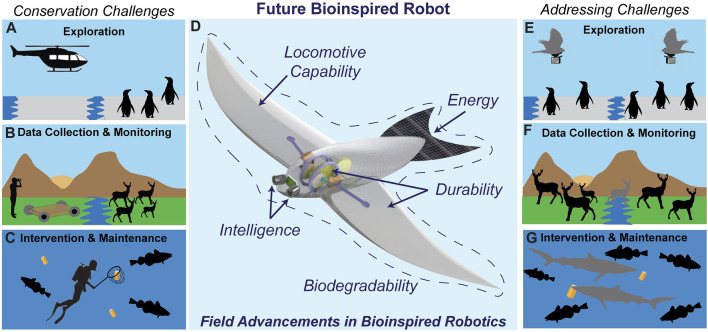
Illustration shows the current challenges associated with conservation efforts and presents future bioinspired robots as an attractive solution to address those challenges in aerial, terrestrial, and aquatic environments. **(A–C)** provide representative use cases of humans and conventional robots involved in conservation efforts and their limitations and negative impacts; **(A)** noise pollution and behavioral change, **(B)** inaccessibility to collect high-quality data, **(C)** inefficiency to collect non-biodegradable waste. **(D)** highlights the operating considerations for the efficient, sustainable, and widespread use of bioinspired robots for conservation using an example of a conceptual bird inspired robot (adapted from (Chellapurath et al., 2021a). **(E–G)** depicts advantages of bioinspired robots (colored gray) in each scenario corresponding to **(A–C)**; **(E)** low noise and environmental integration, **(F)** close proximity high quality data collection/monitoring and environmental integration, **(G)** efficient intervention for waste collection over large spatial scales using multiple bioinspired robots. *Illustrations and Silhouettes taken from Adobe Stock and illustrators V. Deepak, Ignacio Contreras, and Tony Ayling (vectorized by T. Michael Keesey)*.

At present, a majority of conservation efforts are human-led. Human-led exploration, data collection, and monitoring can be risky and is often based on opportunistic sampling and/or use of stationary recording devices (camera and/or sensors) to collect data at regular intervals (Zwerts et al., 2021). Many locations that remain largely uncharted and challenging for humans including cave structures (Candiroglu and Gungor, 2017), the ocean floor (Beck Eichler and Barker, 2020), and extreme cold Arctic and Antarctic regions can provide important information on the ecosystem health. However, such locations present logistical complexities with limited infrastructure, restricted transportation options, and adverse environmental conditions that hinder comprehensive conservation endeavors. A secondary consequence of human led missions is the possibility of harming the environment, disturbing the inhabiting organisms, or influencing the conservation task itself (Figures 1A–C). Using helicopters for biodiversity surveys has been shown to influence the behavior of wildlife in its vicinity (Anderson, 2007) while contributing towards environmental pollution (Figure 1A). Similar expectations hold for automobiles used for wildlife monitoring that can harm small wildlife and the landscape during operation. Intervention and maintenance tasks pose additional challenges since they often require direct human involvement, such as administering medication to animals, resolving human-animal conflicts, or undertaking restoration efforts. The complexity of such tasks require special human expertise and equipment/resources, consequently restricting the scope of their implementation (Boström-Einarsson et al., 2020; Camarretta et al., 2020).

Tackling some of the issues related to human-led efforts has seen the adoption of robotic systems to facilitate in various conservation tasks. Unmanned Aerial Vehicles (UAVs) are used to scan large forest areas to monitor canopy cover while leaving the landscape untouched. They have also been employed to track whales on the ocean surface and fly through unexplored caves that are inaccessible to humans (Hodgson et al., 2013; Zhang et al., 2017; Horton et al., 2019). Autonomous Underwater Vehicles (AUVs) and Remotely Operated Vehicles (ROVs) are used to explore marine life and observe the ocean floor at depths which are risky for manned missions (Sward et al., 2019). Terrestrial robots are deployed to explore and survey landscapes, including active volcano areas and glaciers (Muscato et al., 2012). Overall, these systems have allowed researchers and conservationists to survey larger and more diverse areas, and make more frequent repetitive measurements. Intervention and maintenance tasks have also benefited from the use of robotic systems. Large scale forest and coral restoration has been demonstrated using UAVs and AUVs, however, these applications rely on high quantity of plant seed or coral larva dispersal to increase the odds of tree/coral generation (Dunbabin et al., 2020; Mohan et al., 2021). Hybrid systems such as virtual fences and smart beehives have positively impacted the field of agriculture by providing real-time animal monitoring and reducing human-livestock conflict (Butler et al., 2006; Cecchi et al., 2020).

Despite the wide use of robotic systems to assist in conservation tasks, their conventional design, locomoting mode, and size can make them ineffective in many situations encountered during conservation. Conventional locomoting mode of wheeled robots makes it challenging to cross gaps, move on uneven terrains, and traverse obstacles (Figure 1B) (Gao et al., 2018). Entering canopies for close inspection and sampling or navigating spatially tight aerial environments with conventional multi-rotor UAVs is accident prone, which is in addition to their loud rotor noise that can disturb wildlife (Christiansen et al., 2016). In aquatic environments, propeller driven AUVs or ROVs can suspend sediments from the water bed and trap lifeforms in their slipstreams which can harm aquatic life and contaminate samples during collection (Chellapurath et al., 2021b). Robotic systems with rigid structural components and lack of sophisticated computation and control, limits their ability to perform precise and intricate movements which are required in many intervention and maintenance tasks. For example, delicate handling of the animal for administering medication or instant decision making to resolve a rapidly evolving human-animal conflict situation remain beyond the capabilities of present-day robotic or hybrid systems.

Improving the capabilities of conventional robots in terrestrial, aquatic, and aerial environments has led to researchers drawing inspiration from millions of years of evolution in nature (Sadeghi et al., 2020; Penick et al., 2022), resulting in the establishment of the fields of biomimetics and bioinspired robotics. Biomimetic robotic systems directly incorporate aspects of the morphology, mechanics, sensing or control found in biological systems. Bioinspired robots abstract the fundamental principles behind the form and function of a biological systems to improve their capabilities (Popovic, 2019). Both of these approaches can be implemented in parallel in a robotic system. Recently, several bioinspired robots have aimed to emulate both the physical appearance and the intrinsic dynamics of biological movement, resulting in robust and agile robots (Gravish and Lauder, 2018), that can move, sense, and even look like plants or animals. These new capabilities allow these robots to access different ecosystems and spaces that were not possible before (Savage, 2022). Moreover, it opens up the possibility to operate in close proximity to the local flora and fauna without disturbing it and the environment’s natural state (Li et al., 2019). Altogether, these characteristics make bioinspired robots an attractive candidate to undertake a variety of conservation tasks. A subset of bioinspired robots operating in terrestrial, aerial/arboreal, and aquatic environments are presented in Table 2.

**TABLE 2:** Bioinspired robots used for exploring natural environment with potential application for nature conservation.

Robot	Environment	Description	Organisation	References
Aerial	Robird	- Bioinspired robot that closely resemble bird in appearance and flight behaviour	Aerium Analytics and University of Twente	Folkertsma et al. (2017)
		- Serves as an environment-friendly means of bird control		
Aerial	Robobee	- Inspired by flying insects they can achieve vertical take-off, hovering, and steering	Harvard University	Wood et al. (2013)
		- Can be used for environmental monitoring, search-and-rescue missions, and crop pollination		
Aquatic	Ocean One	- Underwater humanoid robot equipped with a bimanual system and haptic feedback	Stanford University	Khatib et al. (2016)
		- Perform challenging manipulation tasks in inhospitable marine environments		
Aquatic	SILVER 2.0	- Bioinspired underwater hexapod robot inspired by crabs	Scuola Superiore Sant’Anna	Picardi et al. (2020b)
		- Walks and runs on the seabed, recovering trash, sampling sediment, and monitoring marine habitats		
Aquatic	SoFi	- Soft robotic fish that can swim in three dimensions, capable of agile swimming manoeuvres	MIT	Katzschmann et al. (2018)
		- Equipped with cameras to continuously record the aquatic life		
Terrestrial	ANYmal	- Autonomous quadrupedal robot capable of dynamic running and high-mobile climbing	ETH Zurich	Bellicoso et al. (2018)
		- Operate in alpine, forest, underground, and urban environments		
Terrestrial	MIT Cheetah	- Quadrupedal robot that can see and jump over hurdles as it runs	MIT	Seok et al. (2014)
		- Traverses rough terrains, climbs debris-laden stairs, and swiftly recovers from unexpected disturbances		

In this perspective, we highlight the growing field of bioinspired robotics in the context of conservation. We discuss the potential ways in which bioinspired robots can perform exploration, data collection, monitoring, maintenance, and intervention tasks while minimally influencing the natural state of the environment and its inhabitants. We summarize the operating and environmental considerations that must be fulfilled for bioinspired robots to be used as an effective conservation tool. Finally, we present additional avenues that can be explored using bioinspired robots that should further enhance its role as an effective tool to protect and preserve our natural world.

## 2 Future trends in bioinspired robotics benefit nature conservation

We approach the development of bioinspired robots for conservation in two parts. First, the operating considerations that guide its development to ensure its longevity, sustainability, and usefulness in field operations (Figure 1D). Second, the environmental considerations that are dictated by conditions specific to terrestrial, aerial, and aquatic environments which influence the robots interaction with the environment.

### 2.1 Operating considerations

#### 2.1.1 Locomotion and manipulation capabilities

Recent advancements in bioinspired robotics have led to the development of robots that can move and adapt to various environments using different modes of animal-like locomotion (Lock et al., 2013). For example, Salamandra robotica II is a bioinspired robot that can operate in land and water (Crespi et al., 2013). This kind of multimodal capability is extremely relevant for conservation efforts, as many ecological phenomena are interconnected across different environments. Additionally, development of bioinspired robots with specialized locomotory capabilities has allowed navigating challenging environments such as walking on water (Chen et al., 2018), climbing up walls (Spenko et al., 2008), and running on the seabed (Picardi et al., 2020b). These new capabilities can eventually expand the scope of conservation efforts around the world, and introduce bioinspired robots to more hazardous and remote environments to help collect data and perform intervention tasks for conservation. Recent advancements in bioinspired locomotion utilizing animal-like propulsion methods can also enable effective monitoring and engagement with the natural environment, while mitigating issues related to noise pollution (Picardi et al., 2020a), and disturbance to the environment’s natural state (Katzschmann et al., 2018).

The increase in accessibility through enhanced locomotion capabilities will potentially increase the opportunities to perform intervention and maintenance tasks. Intervention often involves robotic manipulators that can delicately handle an organism in its natural setting. Soft robotic arms with tactile feedback from embedded sensors will provide a safer alternative to conventional hard-material robotics, allowing the robots to safely interact with living organisms (Shintake et al., 2018; Liu et al., 2020; Gruber and Wood, 2022).

Overall, developing robots with environment specific locomotion and manipulation capabilities inspired by the inhabiting organisms will allow robots to enter previously inaccessible spaces and interact in novel and more natural ways with their surroundings to perform activities ranging from data collection to intervention and maintenance (Figures 1E–G).

#### 2.1.2 Durability

In conservation activities, exploration and data collection often require using robots repeatedly in rugged and harsh conditions which can lead to wear and tear on their structures. Furthermore, operating in harsh environments increases the risk of experiencing accidents and/or failures. The use of adaptive structures, high-performance materials, and self-cleaning mechanisms can increase the durability of these robots. Adaptive structures, which can vary in shape and/or stiffness, can better withstand changing environmental conditions and tasks, reducing the stresses on the body (Cully et al., 2015; Khaheshi and Rajabi, 2022). They also provide added functionality and cost-effectiveness. High-performance materials can provide strength, resilience, and corrosion resistance to the robot’s structural components (Pan et al., 2020). The incorporation of flexible electronics can allow the robot to withstand physical stressors from the environment and maintain performance and durability to extend its operating window (Huang et al., 2019; Phillips et al., 2022). The use of self-healing materials will further increase the robot’s operational window in unpredictable environments and bring it closer to achieving autonomous field operation. Eventually, these material considerations can increase the robot’s durability, and potentially reduce costs and electronic waste, paving the way for their widespread and long-term use in conservation (Tan et al., 2021; Terryn et al., 2021).

It is important to carefully evaluate different approaches and weigh their trade-offs when selecting materials and mechanisms to improve the durability of conservation robots. For instance, while adaptive structures can enhance robustness and functionality, they may also increase costs. Similarly, self-healing materials can improve the operational window, but their advanced and costly manufacturing process can pose a challenge. To determine the most appropriate option, one must consider the specific needs and requirements of the conservation activities, as well as the environmental conditions and potential risks associated with operating the robots in those conditions.

#### 2.1.3 Intelligence

Biologically inspired intelligence involves incorporating biological strategies, mechanisms, and structures into robotics research and has been investigated as a means to develop more efficient methodologies and technologies for addressing existing challenges (Li et al., 2021). Integrating biologically inspired intelligence into robots intended for use in exploration, data collection, and monitoring can impart characteristics such as adaptability, robustness, versatility, and agility. These characteristics are crucial to safely navigate complex unknown environments. They can also enable smooth transitions between locomotion modes when moving from one environment to another (e.g., aerial to arboreal or terrestrial to aquatic) (George Thuruthel et al., 2021; Biewener et al., 2022; Miki et al., 2022).

Moreover, the field of neuromorphic computing and engineering, which involves creating computational systems based on biological structures, has made significant advancements and has the potential to enhance bioinspired robots’ real-time interaction with the physical world (Zhao et al., 2020; Christensen et al., 2022). The development of controllers that can adapt to damages and morphological changes in bioinspired robots will be a significant leap forward in the exploration of hazardous environments. They can enable the control of shape-morphing multi-modal robots, which can change their form and functionality to better navigate and operate in different conditions.

Based on the conservation task, robots should be able to exhibit collective behavior to perform tasks beyond the capabilities of a single individual, with minimal explicit communication (Dorigo et al., 2013; McGuire et al., 2019; Berlinger et al., 2021). This type of collective intelligence, known as swarm intelligence (Schranz et al., 2021), is particularly useful for studying spatio-temporal phenomena such as wastewater plumes, oil spills, convection, and biologically active layers that require simultaneous sampling at multiple locations (Schill et al., 2018). Swarm systems, unlike single robot systems, can continue functioning even if individual robots fail or need to be removed, as they can adapt to changes in the number of robots using only local communication (Jaffe et al., 2017). Swarm robotics has already demonstrated its effectiveness within the field of high precision agriculture (Kondoyanni et al., 2022). Initiatives such as ‘Mobile Agriculture Robot Swarm’ (Blender et al., 2016) have harnessed the capabilities of swarm robots to execute various intricate farming tasks, typically associated with human involvement. This utilisation has led to enhanced crop yields and a decreased ecological footprint. Analogously, comparable swarm robotics systems hold promise for monitoring, intervention, and maintenance tasks aimed at the preservation of natural ecosystems. For example, in the project, CoCoRo (Schmickl et al., 2011), a swarm of robots was designed to navigate though underwater habitat while coordinating the swarm members through bioinspired and biomimetic algorithms. Similar to a school of fish, they engaged in the exchange of information to monitor, maintain, and harvest resources in the underwater environment.

#### 2.1.4 Energy

One of the main challenges in achieving full robot autonomy in field operation is the limited capacity of energy storage systems, particularly battery cells, which have not undergone significant changes in design and efficiency. Robots with traditional lithium-ion batteries must be frequently retrieved to replace/recharge the battery followed by redeployment, limiting the duration and economic feasibility of field operations, especially in remote and hostile environments. This is particularly challenging in microrobots which deal with low battery life resulting, at times, in the use of a tether (Lok et al., 2017). Alternative energy dense options using hydraulic fluids could facilitate increased energy density, autonomy, efficiency, and multi-functionality in future robot designs (Aubin et al., 2019). Eventually, robots in field operation should be capable of harvesting energy from renewable sources and/or receive energy wirelessly to supplement or replace their on-board energy source. These capabilities aim to reduce the environmental impact of electronic waste and significantly extend the robot’s operational window (Liang et al., 2022). For example, EcoBot III, which has an organic digestive system to power itself, demonstrates the advancements in energy harvesting capabilities in robots. (Ieropoulos et al., 2010).

Efficient and intelligent robot controllers can significantly improve a robot’s autonomy and working window by reducing energy consumption and optimizing the decision-making process. Consequently, robots can operate for extended periods without requiring frequent battery replacements, leading to cost savings and improved operational efficiency (Li et al., 2020).

#### 2.1.5 Biodegradability

Successful retrieval of the robot after completion of its task is critical; we propose bioinspired robots as a tool to remedy the harmful environmental impacts rather than an enabler of environmental degradation. Swarm robots exemplify the importance of biodegradibility where multiple robots are in use to perform a task and the unsuccessful retrieval of one or more individual robots can negatively impact the environment. The development of small fully biodegradable robots can allow their deployment in vast numbers to inaccessible locations for conservation tasks before safely biodegrading into the environment (Kim et al., 2022; Rumley et al., 2023).

A variety of biodegradable materials including cellulose/carboxymethylcellulose, polylactic acid (PLA), and polypropylene fumarate (PPF) can be utilized to create biodegradable structure of the robots which can degrade after accomplishing their specified mechanical function in the field (Sethi et al., 2022). However, to achieve complete biodegradability, electronics and energy source must also be made biodegradable. Though advances have been made in the field of biodegradable electronics (Tan et al., 2016), the developments on a biodegradable energy source like Microbeal Fuel Cells (MFCs) remains extremely challenging. MFC-equipped robots present several challenges while operating in a natural environment, which include the need for nutrient rich liquid feedstocks (Ieropoulos et al., 2010), low power output, and vulnerability to the infection of bacteria or fungus. This limits their operational scope and confines the usage to slow or passive tasks. However, recent progress on MFCs, particularly in reactor configuration and system architecture, separator, and cathode catalyst is promising to achieve the goal of completely biodegradable robots (Gajda et al., 2018; Winfield et al., 2019).

An alternative sustainable approach involves substituting the conventional digital sensors on the robot with bioindicators for the purpose of monitoring and assessing environmental conditions. Bioindicators encompass living organisms like plants, plankton, animals, and microbes, which are employed to evaluate the ecological wellbeing of the natural surroundings (Holt and Miller, 2011). For instance, in the project Robocoenosis, Zebra mussels and Daphnia were used as living sensors to monitor natural underwater environments (Rajewicz et al., 2022). This strategy reduces reliance on non-biodegradable components within the robot’s sensing system. Furthermore, analysing the bioindicators in the environment via camera visuals from the robot can minimise the dependence on other traditional sensors. For instance, urban areas can incorporate lichens or fungi onto building walls. These biological indicators serve to reflect the air quality within the city (Matos et al., 2019; Ilgün et al., 2022). Employing an aerial robot with an onboard camera to assess the coloration of these structures can provide insights into the air quality.

### 2.2 Environmental considerations

Robots functioning in a natural environment require regular maintenance tasks like cleaning, lubrication, and inspection, along with the repairing or replacing of robot’s components that have experienced wear and damage. Furthermore, different ecological environments impose specific technological challenges for the robot’s optimal functionality (Figures 1A–C). These specific challenges in bioinspired robots to foster conservation in terrestrial, aerial/arboreal, and marine environments are described below:

#### 2.2.1 Terrestrial

Terrestrial conservation tasks require the robot to perform in urban, rural, and natural environmental conditions. Successful operation would require a combination of locomoting and perceiving capabilities that allow the robot to adapt to different terrains and surface properties (hardness, slipperiness, or irregularities). The integration of proprioception and exteroception coupled with the capability to move like animals can make robots versatile and effective on substrates such as sand, snow, and vegetation. A recent study that incorporated these principles in a legged robot has shown the potential for successful navigation in diverse environments, including alpine, forest, underground, and urban settings (Miki et al., 2022).

One of the significant challenges facing terrestrial robotics is the problem of path planning (Figure 1B). Path planning by building a map in a distributed manner by a swarm of legged robots is one of the solutions to this challenge (Ramachandran et al., 2020). The integration of terrestrial robots with aerial robots presents another promising solution to this challenge. By utilizing visual mapping information provided by aerial robots, terrestrial robots can plan traversable paths and achieve their desired goals with increased efficiency and effectiveness (Käslin et al., 2016).

#### 2.2.2 Aerial/arboreal

Operating in aerial and arboreal environments requires counteracting the pull of gravity while performing the conservation task at hand. In addition to flapping robots (Yousaf et al., 2021), developing bioinspired robots that can takeoff from and move on uneven vertical substrates, glide, and perch will significantly expand the scope of conservation efforts to include entering forest canopies, collecting samples, and easily transition from arboreal to aerial environments and vice-versa (Figure 1D). Moving on vertical substrates will allow close inspection and maintenance tasks while eliminating human risk (Spenko et al., 2008). Unlike UAVs, perching and grasping will reduce the reliance on lift generation and thus energy expended. It will also enable the robot to hold position with minimal control effort which is often required for data collection (Roderick et al., 2021; Siddall et al., 2021; Chellapurath et al., 2022). Glide capabilities, like in animals (Zhao et al., 2019; Khandelwal and Hedrick, 2022), can increase the flight time by reducing the dependence on powered flight, reduce noise pollution and make them more robust to aerial perturbations.

#### 2.2.3 Aquatic

In an underwater environment, the robot experiences additional forces associated with water, such as buoyancy, hydrodynamic drag, and added-mass effect, which must be taken into account during the design, control, and maintenance of the robot (Picardi et al., 2020b; Katzschmann et al., 2018). Additionally, the pressure experienced by an object increases by 1 atm for every 10 m of depth, requiring all electronic components in a robot to be sealed in rigid watertight canisters, limiting the flexibility of designing compliant and soft bioinspired robots for use at extreme depths. Moreover, the underwater structures require high maintenance as they are highly prone to corrosion and fouling.

Recent advancements in technology have led to the development of an untethered soft robot for deep-sea exploration, utilizing a self-powered design inspired by the structure of a deep-sea snailfish. The delicate electronic components are embedded and distributed within a soft silicone material which eliminates the need for pressure-resistant cases. This innovative design holds potential for future deep-sea exploration and research (Li et al., 2021).

Underwater visibility issues are also encountered by robots. However, aquatic creatures have adapted sensory mechanisms to overcome these challenges. Seals, for instance, can detect and monitor herrings up to 180 m away by utilizing their wavy whiskers (Zheng et al., 2021). In addition, fish possess mechanosensory lateral-line systems that allow them to perceive and detect their hydrodynamic and physical surroundings (Mogdans, 2019). These natural mechanisms can serve as a source of inspiration for the development of sensor systems in underwater robotics.

Moreover, in the aquatic ecosystem, biological functions from nutrient cycle to energy transfer in food webs see a strong coupling between pelagic and benthic zones (Griffiths et al., 2017). To have a broader understanding on ocean and freshwater ecosystems, data has to be gathered from both zones. Moreover, there is also a need for precise close-range 3D acquisition of benthic environment (Bruno et al., 2011), for example, monitoring the growth of coral reefs. Hence, together with pelagic robots (Yu et al., 2016; Morimoto et al., 2018; Romano et al., 2022), focus is also needed on robots that can perform exploration and monitoring in the benthic region (Picardi et al., 2020b).

## 3 Discussion

Conservation efforts are critical to combat the deteriorating health of our planet. In this perspective, we discuss the development of bioinspired robots as versatile agents that can significantly expand the scope and effectiveness of conservation efforts around the world while minimizing the negative impact on organisms and the environment’s natural state.

Achieving a future where bioinspired robots can perform conservation tasks of exploration, data collection, intervention, and maintenance requires developing novel capabilities, akin to how animals move, sense, and interact in the natural world. Such capabilities can be realized through the concept of physical artificial intelligence, i.e., co-evolving the morphology, actuation, control, and sensing of physical systems can provide them with capabilities to perform tasks akin to intelligent organisms (Miriyev and Kovač, 2020). Moreover, the development of such capabilities can benefit from studies on the biomechanics, ecology, and sensing in animals which provide insights into the physical and behavioral basis of how organisms adapt, move, and interact in different ecosystems (Jackson et al., 2016). Overall, the understanding gained from organisms coupled with the concept of physical artificial intelligence creates a paradigm for researchers on ‘how’ to create versatile robots that are skilful to manipulate unknown objects, move in unpredictable complex environments, and interact with surrounding organisms. (Kanko et al., 2021; Bicer et al., 2022).

However, the development of robots with animal-like capabilities alone cannot lead to their effective and widespread use in future conservation efforts. Here, we present additional considerations for the robot that include interdisciplinary research collaboration, practicality in research and field operations, and applicability as interactive agents for conservation.

### 3.1 Collaboration

Tight collaborations between engineers, biologists, and conservationists is critical to develop bioinspired robots that function like animals and can gather relevant data that is required for the conservation task. Through such collaborations, roboticists/engineers can build bioinspired robots meeting the specific needs of conservation biologists, and in turn conservation biologists can provide valuable expertise on the behaviors, habitats, and ecosystems that the robots will be interacting with (Berger-Tal and Lahoz-Monfort, 2018; Schulz et al., 2023). Furthermore, wherever possible, involving the local communities during the development, maintenance, and troubleshooting process of the bioinspired robot will ensure that conservation efforts are sustainable and effective in the long-term without assistance from researchers.

### 3.2 Practicality

Bioinspired robots must be a cost-effective proposition to significantly contribute towards conservation, especially since conservation projects often have limited budgets. A purpose built bioinspired robot for a conservation task should be favored over a general purpose robot since the former will minimize the hardware and software requirements and drastically reduce the cost (Byagathvalli et al., 2021). Additionally, the use of easily available components, low cost fabrication/manufacturing techniques, and modular and scalable designs can further reduce the robot’s cost and upkeep (Thomas and Gilbert, 2014). For example, a modular design can help with troubleshooting to ensure rapid turnaround times between repair and redeployment of the robot in field operations (Brooks et al., 2005; Thomas and Gilbert, 2014). Finally, utilizing and publishing open-source designs and technology will enable conservationists and researchers to readily adapt the designs for other research, environmental, and conservation purposes (Kulkarni, 2019).

Commercial bio-hybrid implementations such as smart beehives and virtual fences are testament to the potential and practicality of technology in facilitating conservation tasks (Jachowski et al., 2014). Bioinspired implementations have also been developed such as ‘Spot’ from Boston Dynamics and ‘ANYmal’ from ETH (Hutter et al., 2016), demonstrating that such technology is readily being adopted for commercial applications and is not limited to academia. However, these robots are expensive, making them out of reach for most conservation projects. In the future, cost reduction through commercial use and scaling up production can make direct purchase from companies a viable option.

Overall, accounting for collaboration and practicality during the development phase of the robot considerations can further expand the potential impact of the bioinspired robot by facilitating its wider adoption among the conservation and research community.

### 3.3 Bioinspired robots as interactive agents for conservation

With capabilities that allow bioinspired robots to move, sense, and interact like animals, they can be deployed in close proximity to wildlife and enter previously inaccessible environments to collect data or perform intervention and maintenance tasks (Figures 1E–G). This versatility extends their use beyond these conventional conservation tasks, offering researchers the opportunity to explore diverse and novel applications.

Nobel laureates, Konrad Lorenz and Nikolaas Tinbergen conducted pioneering experiments using mechanical dummies that look like animals to study animal behaviour in controlled settings (Burkhardt Jr, 2014). Their studies laid the foundation for integrating biomimetic devices into living systems (Webb, 2000; Krause et al., 2011). Recent advancements in bioinspired and biomimetic robotics have facilitated the development of artificial systems that can interact with living systems in increasingly creative ways. The interactions between these artificial devices and living organisms are evolving into influential bio-hybrid agents, with the potential to significantly contribute to ecosystem conservation efforts (Ilgün et al., 2021). For example, biomimetic fish robot, Robofish, which was used to investigate the collective behaviour of fish like recruitment and leadership (Faria et al., 2010). In the LEURRE project, an integration of American cockroaches and miniature insect-like robots known as Insbots was established, creating an experimental mixed society. The fundamental aim of this initiative was to demonstrate the possible control of these mixed societies, which is a key challenge in many scientific fields, including ethology (Caprari et al., 2005). Recently, researchers were successful to study the dance-following behaviour in bees using a robotic bee called ‘Robobee’ (Landgraf et al., 2018).

These social integration of robots into animal societies is referred as ‘organismic augmentation’ (Schmickl et al., 2021). Such augmentations can also create artificial ecological interactions via inter and/or intra species communications to influence the behaviour of the animals at their society level, which ultimately affects the ecosystem in which the society is embedded (Bonnet et al., 2019; Lazic and Schmickl, 2021). This new paradigm of ‘ecosystem hacking’ (Stefanec et al., 2022) via organismic augmentation can positively effect the ecosystem stability or at least slow down the ecosystem decay.

Additionally, the ‘interactive’ bioinspired robots (Datteri, 2020) can potentially be used to train captive animals before their reintroduction into their natural habitats. Moreover, these robots can mitigate human-wildlife conflicts, such as using robot wolves instead of electric fences to safeguard agricultural fields from wildlife (Bendel, 2022), or utilizing bird-inspired flapping robots to deter birds from congregating near airports during flight operations (Patel and Rughani, 2022). Bioinspired robots can also be used to non-invasively study animal behavior and locomotion in the wild (Datteri, 2020; Romano et al., 2020; Li et al., 2022), as shown in studies on bat echolocation (Bou Mansour et al., 2019) and locust jumping direction (Romano et al., 2019). Ethorobotics, a growing field of biorobotics, proposes leveraging robotic replicas as an innovative approach for exploring animal behavior (Romano et al., 2020), like social learning in vertebrates (Romano et al., 2021) and zebrafish shoaling (Ruberto et al., 2016).

The valuable insights gained through these interactions can help conservation biologists better understand their animal of interest and assist them in designing tailored and effective conservation strategies.

## 4 Conclusion

Leveraging advancements in design, materials, intelligence, and energy harvesting is leading to the rapid evolution of bioinspired robots. Specifically, improved locomotory capabilities allow them to overcome operational challenges in terrestrial, aquatic, aerial, and arctic environments. The animal-like appearance and behavior allow easy-integration into the natural environment and interact with the surrounding while minimizing disturbance to inhabitants and preserving the environment’s natural state. Altogether, these capabilities make them versatile agents for future conservation efforts by overcoming current limitations of human-led conservation activities.

Through this perspective, we provide a framework for researchers to develop bioinspired robots with animal-like capabilities that have the potential to revolutionize conservation efforts around the world. These robots can offer sustainable and effective ways to explore uncharted environments, carry out ecological field missions, facilitate data collection and monitoring in extensive, standardized, and repeatable ways, and carry out intervention and maintenance tasks in an efficient and precise manner.

To further strengthen conservation efforts, we highlight the importance of fostering conservation through interdisciplinary collaboration, considering the practicality in research and field operations, and exploring diverse applications. We hope that this piece will encourage future researchers to design and develop bioinspired robots catering to the pressing issue of conservation that is critical to save our planet from rapid biodiversity loss and improve the overall wellbeing of all its inhabitants.

## Data Availability

The original contributions presented in the study are included in the article/supplementary material, further inquiries can be directed to the corresponding author.
